# Short term ex vivo storage of kidneys cause progressive nuclear ploidy changes of renal tubular epitheliocytes

**DOI:** 10.1038/srep10341

**Published:** 2015-06-03

**Authors:** Huaibin Sun, Jun Tian, Wanhua Xian, Tingting Xie, Xiangdong Yang

**Affiliations:** 1 Department of Hemodialysis, Qilu Hospital, Shandong University, Jinan 250012,China; 2 Department of Nephrology, Qilu Hospital, Shandong University, Jinan 250012, China

## Abstract

In renal transplantation, there has been considerable success, mainly in term of post-transplant graft function. However, upon closer scrutiny, it is known that severe dysfunction, including persistence of renal failure is seen after transplantation. The major condition that potentially cause significant lesion may be hypothesized to be related to the hypothermic approach to storage. To systematically examine these issues, we stored mammalian (sheep) kidneys in UWS at 4 °C for four different time points (0, 1, 3 and 6 hours). We obtained renal histological sections and examined tubular architecture as well as nuclear characteristics of tubular epitheliocytes. The results of our preliminary investigations suggest that there are temporal changes of tubular epitheliocytes, as well as genomic changes. These changes were also seen in tissues stored at room temperature. Our observations suggest the need for additional studies for redesigning of improvised storage solutions. Pilot studies using Celsior also revelaed similar kind of nuclear changes, suggesting that storage conditons are contributory, including perfusion versus static conditions. The results may explain persistence of tubular injury several days after orthotopic transplantation, and may potentially be contributory to delayed graft function (DGF).

There have been several paradigm shifting discoveries related to organ transplantation^1^,^2^,^3^,^4^,^5^,^6^,^7^. Chief among them includes the innovations that pertain to organ storage ex vivo^8^,^9^,^10^,^11^,^12^. These storage solutions include Celsior, University of Wisconsin solution and HTK^13^,^14^. These solutions have been designed mainly with respect to maintaining cellular metabolic needs *in vitro*. However, in addition to slowing metabolic enzymatic processes, an additional biophysical parameter is incorporated during storage conditions. Namely, the organs are stored in cold solutions. The chief rationale for storing under cold condition involve prevention of ischemia-reperfusion injury post transplantation.

In the clinical setup, these cold conditions are taken for granted. Scant pathophysiologic examinations have revealed that cold condition can actually cause cellular damage^15^. Cytoskeleton forms the main micro architecture of cells, and recent evidence has built-up which shows cytoskeletal disruption as a result of prolonged ex vivo storage, possibly attributable to degeneration due to tepid temperatures^16^,^17^. For example, this study showed that hypothermic storage preservation causes degradation of important cytoskeletal components in kidney tubules, which contributed to the injury at rewarming^18^. Progressively prolonging the cold storage time of isolated renal tubules in University of Wisconsin solution caused disassembly of ezrin and the enzymatic Na/K adenosine triphosphatase proteins from their cytoskeletal adhesions. The sublamellar structural protein, fodrin, was degraded by calpain hydrolysis. Time-dependent disorganization in tubule membrane function (organic cation transporter-1 transport activity) was also demonstrated during the cold preservation. Cold preservation decreased the total tubulin as well^18^.

In the renal transplantation clinical scenario, there has been considerable success, mainly in term of post-transplant graft function. However, upon closer scrutiny, it is observed that severe dysfunction, including persistence of renal failure is seen after transplantation^19^. While a portion of this may be causative due to original pre-renal cause contributions to the compromise of renal function, a greater proportion may actually result from graft dysfunctions acquired during ex vivo storage. The major condition that potentially cause significant lesion may be hypothesized to be related to the hypothermic approach to storage.

In order to systematically examine these issues, we stored mammalian kidneys in UWS at 4 °C for four different time points (0, 1, 3 and 6 hours). UWS, Celsior and HTK solutions have been used for kidney storage. We focused on only one solution, as comparisons have revealed that these solutions do not show major changes during ex vivo storage^20^,^21^,^22^,^23^,^24^. We obtained sections and examined tubular architecture as well as nuclear characteristics of tubular epithelial cells. In order to validate the findings, we stored kidneys in UWS at room temperature (25 °C) for 6 hours and compared the changes. Furthermore, we also stored freshly obtained high grade renal clear cell cancer biopsy samples and stored either in the cold or at room temperature to examine whether nuclear ploidy changes occurred in these cancer samples. The results of our preliminary investigations suggest that there are temporal changes of tubular epitheliocytes, as well as genomic ploidy changes which may explain persistent of tubular injury several days after orthotopic transplantation, and may potentially be contributory to delayed graft function (DGF). These changes may be dependent on the type of storage solution used for ex vivo storage, and provides a rationale for improvising current standards of storage solution. The time period we chose conform to the standard time periods currently used in protocols for storage of beating heart donor (BHD)-donated kidneys.

## Materials and Methods

### Ethics statement

All experimental procedures were conducted in conformity with institutional guidelines for the care and use of laboratory animals, and protocols were approved by the Institutional Animal Care and Use Committee of Qilu Hospital, Shandong University, China. Human cancer tissue samples were processed after obtaining explicit consent and approved by Institutional Review Board. Written informed consent was obtained from all subjects.

### Materials used in the experiment

University of Wisconsin solution was prepared in-house according to available proprietary formulae. Chemicals were obtained from Sigma-Aldrich (St. Louis, MO). Celsior solution was also used for storing kidneys in a small series of pilot experiments to establish whether the trend of changes seen in UWS storage conditions is observed in other storage solutions as well. 3 kidneys in each group were stored for 6 hours at both 4 and 25 °C.

### Kidney and human cancer tissue sample procurement and storage

The study was conducted in thirty male sheep, each weighing 30–35 Kg, in accordance with approved animal protocols. Animals were anesthetized by fluothane. Whole kidneys were dissected out by a extraperitoneal lumbar incision during beating heart condition, and then stored in UWS for 0–6 hours at 4 (n = 20) and 25 °C (n = 10). After nephrectomy, the isolated kidneys was flushed with the balanced solution. Kidney biopsies were periodically obtained.

Human cancer tissue samples representing high grade renal cell clear carcinoma were obtained in collaboration with Urological Surgery section. Samples from five independent patients were obtained from the current study. These tissue samples were examined because of their inherent high grade nuclear dysplasia and whether these deteriorated upon ex vivo storage. These findings lend credence to the findings of the current study regarding change in genomic ploidy upon ex vivo storage of normal kidneys.

### Staining and imaging

Haematoxylin and eosin (H&E), Giemsa and Feulgen staining of 7 μM renal cryosections obtained from timed kidney biopsies of all tissues were performed. Tissues were cryoprotected with OCT media prior to obtaining sections and each slide therafter was fixed in formalin prior to staining. Images were viewed immediately after staining and images obtained using appropriate filters using a Zeiss upright microscope. Uniform staining protocols were used in order to make comparisons of ploidy changes under different storage conditions.

### Quantitation of cellular damage

Acquired images were archived and examined for different histological parameters. Quantitative estimates of nuclear aspects were obtained using the softwares ImageJ, Metamorph and PAX-it. Pixel aspect ratios were standardized prior to performing quantitative morphometry. Integrated gray value densities, nuclear shapes, feret diameter and eccentricity were obtained to evaluate changes in global DNA concentrations and nuclear shapes, which is independent predictor of temporal nuclear changes. 32 bit grey scale images were pseudocolored with ImageJ to obtain images with higher contrast, as well as demonstrate changes of progression from open-faced nuclei to further chromatin condensation.

### ATP Bioluminescence assay

ATP contents were assayed in kidney biospies by using 50 mg of renal lysates. Tissue homogenate was stored in perchloric acid and neutralized with KHCO_3_ solution prior to centrifugation and siphoning off the supernatant. All experiments were performed on ice bucket. Protein assays were performed for normalization. ATP assay was performed by a bioluminescence method (Perkin-Elmer).

### Statistical Analyses

Data were expressed as means ± SEM. Analyses of variance was used to compare differences between multiple groups.

## Results

**Figure 1:**
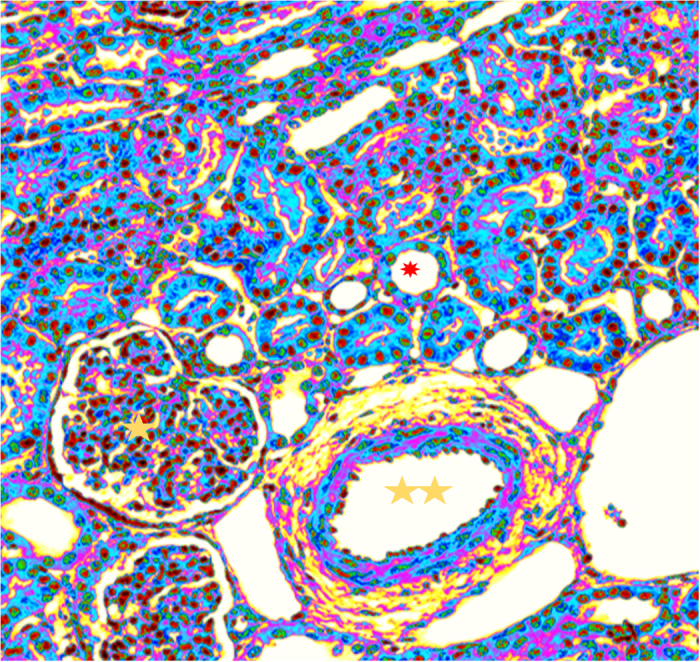
Renal section obtained after 6 hrs of storage in UWS The H&E section has been pseudocolored based on pixel intensities on a RGB scale, with red indicating higher optical intensities of the staining. Computer manipulation of image was performed using ImageJ. No gamma chanes were made during image capture and the intensities are representative of the original stain. Note that most tubular nuclei are red in appearance. These nuclear condensation are also seen in most nuclei in glomeruli, as well as endothelia. Red asterisk, tubular lumen; yellow single asterisk, glomerulus; yellow double asterisk, arterial lumen.

Haematoxylin and eosin staining and quantitative DNA staining revealed progressive nuclear changes. A pseudocolored image of a field of renal section after 6 hours of storage in cold UWS solution is shown in Fig. 1. A full range of color coding showing intensity of staining reveals that most nuclei were condensed in appearance. These were seen not only for nuclei of the tubular epitheliocytes, but also multicellular nuclei within the glomeruli as well as endothelium of a small-artery in field view. We decided to further examine the tubular epitheliocytes, as they form the main cellular components of the urine-producing tubular function.

**Figure 2:**
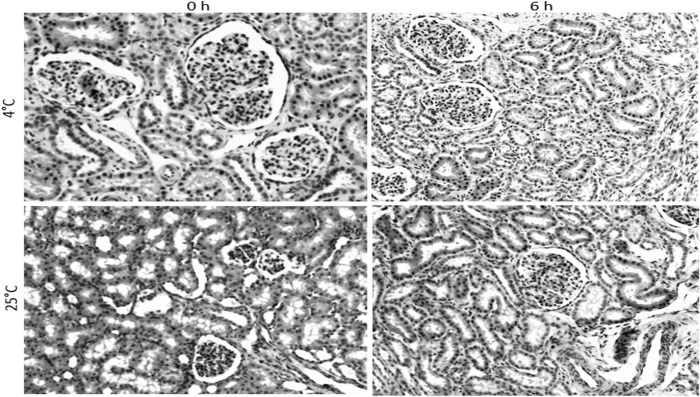
Nuclei of different tissue components of renal sections show nuclear condensation at 6 hrs of storage in UWS, both in cold as well as room temperature H&E stained sections are shown. Scale bars, upper left panel, x200, all others, x100.

The nuclear condensation of tubular epitheliocytes (as well as other renal cellular components) seen after 6 hours of storage of kidneys in UWS under cold conditions were also observed in kidneys stored at room temperature at about 25 °C (Fig. 2). These observations suggest that the nuclear condensation is probably not dependent on the temperature of storage but rather an ongoing degenerative process related to non-perfusion of the organs and probably may be prevented by tweaking the composition of the solution, rather than modulating temperature of storage. All renal sections were examined blindly by a nephropathologist in order to confirm the findings and were scored independently (1, <10% condensations in individual epitheliocytes in high power field that showed at least four complete tubules; 2, <50% condensation, 3 > 50% condensation). The lack of changes in the higher temperature (room temperature) group is an intriguing observation, as ambient storage conditions are known to be supportive of higher levels of HEP (high energy phosphate) synthesis. In fact, our preliminary analyses demonstrated increased HEP levels of total renal lysates (normalized to tissue mass) in the 25 °C group in comparison to the 4 °C UWS-storage group (data not shown). These observations suggest that the observed genomic changes are at least not related to the availability of high energy phosphates by gearing a shift from a suspended of metabolism at cold storage conditions to a more aerobic and stimulated metabolic state of storage of ambient conditions. Obviously, these and other validatory findings definitely also lend evidentiary support to the observations that the nuclear condensation changes are not merely artefactual, resulting from random events. Staining protocol, incluing tissue fiation, were standardized across all specimens. We also ran a pilot series of experiments in which we stored the renal tissues in Celsior, both at low and ambient temperature conditions and observed the same changes as seen in the UWS storage group (data not shown). Though this current study cannot make statistical comparisons of nuclear changes between UWS and Celsior storage because of inadequately powered pilot experiments, it clearly establishes that the trends of nuclear changes seen in UWS solution is not an isolated phenomenon but rather a distinct biological observation.

**Figure 3:**
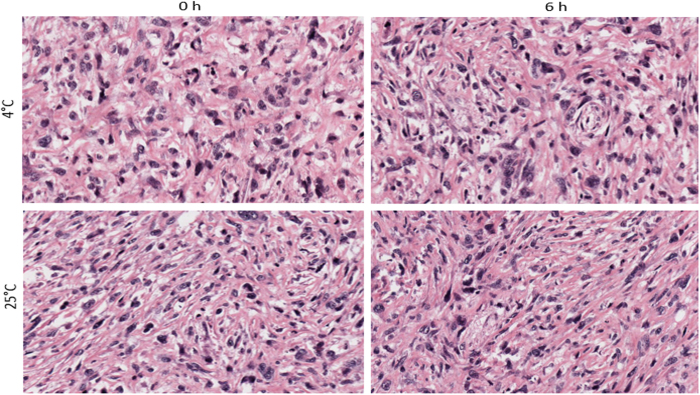
Panoramic nuclear condensation is seen in human renal clear cell carcinoma sections stored in UWS for 6 hrs at 4 and 25 °C Cancer tissue samples were of high grade dysplasia to start with and examination of greater than 25 medium power fields showed progressive nuclear condensation with short term storage of 6 hrs, both in the cold as well as ambient temperature. This suggests storage factors (temperature, as well as composition of storage solution) as important factors in causing degenerative nuclear changes during ex vivo storage; H&E staining.

In order to validate our findings, we stained and examined nuclear condensation in tissue samples of human renal clear cell carcinoma. To start with, many nuclei showed condensation and high grades of dysplasia, and after storage of these tissues in UWS, both at room temperature as well as in the cold, almost all nuclei showed progressive condensation (Fig. 3). These control tissues further help validate our observations that ex vivo storage leads to nuclear changes in short term period during storage in conventional solutions like UWS.

**Figure 4:**
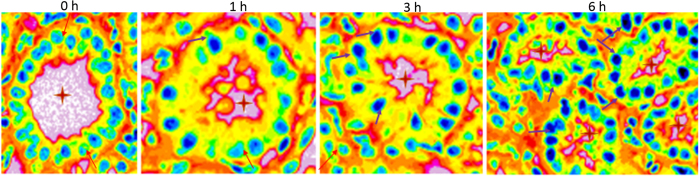
Pseudocolored images of renal tubules showing progressive changes in epitheliocytes, luminal shape and nuclear appearance H&E sections were converted to 32 bit prior to pseudocolor conversion in ImageJ to show enhanced contrast, luminal and nuclear boundaries and contours. Note the progressive changes from open-faced (light sky blue) to close-faced (dark navy blue) condensed nuclei. The four time points are 0, 1, 3 and 6 hours respectively (panels from left to right). Note the progressive temporal crenation of lumen (red star within lumina). Red arrows, open faced nuclei; purple arrows, condensed nuclei.

There was progressive subtle changes in the tubular epitheliocytes (Fig. 4). At time 0h, just after organ retrieval, the tubular epitheliocytes were uniform appearance, with normal orientation for luminal and basal sides. Also, the lumen appeared roundish and regular in appearance. With progressive storage *in vitro*, the shapes of tubular epitheliocytes started showing changes, with loss of the pyramidal appearance of the cells. The lumen also showed changes and lost the regular rounded appearance. Most interestingly, the nuclei showed progressive changes, which prompted us to examine these changes in more details.

**Figure 5:**
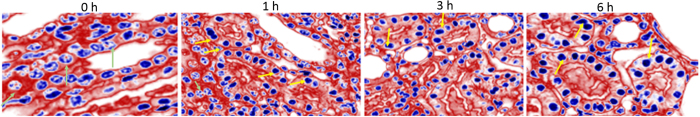
Higher power images showing nuclear changes after progressive storage of kidneys ex vivo under cold conditions H&E sections were converted to 32 bit prior to pseudocolor conversion in ImageJ. The four time points represented are 0, 1, 3 and 6 hours respectively (panels from left to right). Green arrows, open faced nuclei; yellow arrows, condensed nuclei.

**Figure 6:**
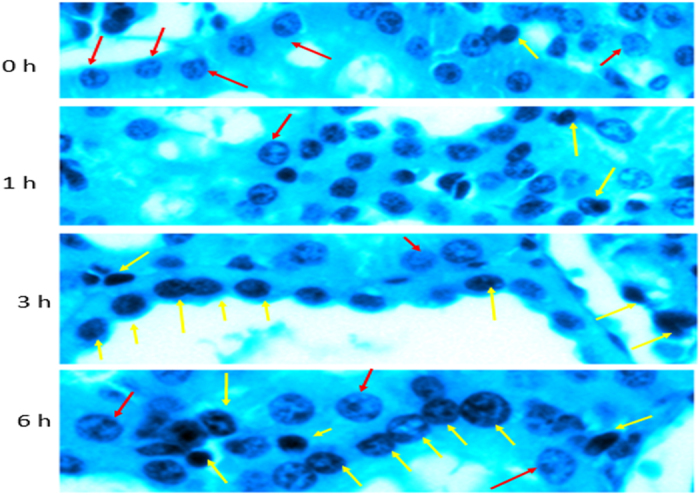
Details of nuclear changes of renal tubules during 0-6 hours of storage in vitro in cold conditions. Higher magnifications reveals the heteromorphic changes. Feulgen staining was used to obtain integrated optical densities to obtain quantitative estimates of nuclear DNA composition. Images were pseudocolored in ImageJ. Non-linear changes were not performed with these images where quantitative nuclear morphometric parameters were estimated. Condensation of nuclei is visualized as progressively dense blue stain, as well as nuclear condensation. The four time points represented are 0, 1, 3 and 6 hours respectively (panels from top to bottom). Red arrows, open faced nuclei; yellow arrows, condensed nuclei.

**Figure 7:**
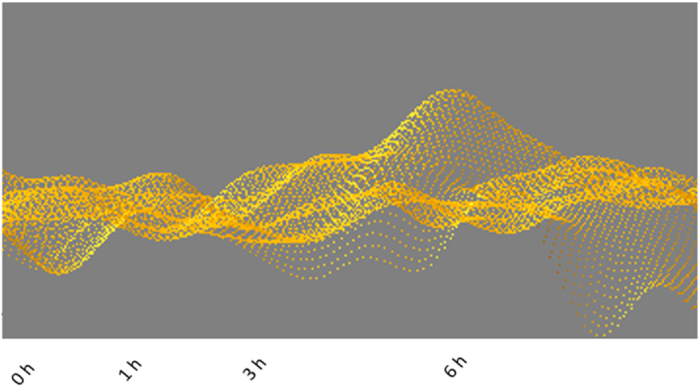
Ploidy mapping of nuclei at different time points The heterogeneity and variance reflects the changes of nuclei, which is seen more at 6 hrs of storage. Feulgen staining was performed prior to imaging the integrated nuclear optical densities. Uniformity of staining was strictly adhere to, so as to facilitate uniformity of staining and validating the comparisons of the quantitative data. A value of one corresponded to nuclear diploidy. One thousand nuclei from 5 indpeendent samples were examined and plotted at each time point examined.

The nuclei were heterochromatin, open-faced at the time of harvesting the kidney (Fig. 5). However, progressive cold storage resulted in drastic changes of the nuclear appearance. Most importantly, several nuclei showed condensed chromatin and disruption of the open-faced appearance, suggesting progressive alterations of nuclear metabolism (Figs. 5 and 6). Nuclear shapes were also changed, including disruption of cellular boundaries of many epitheliocytes, suggesting apoptosis. Genomic DNA changes were examined, which showed enhanced ploidy and wide variation upon 6 hr storage under cold conditions (Fig. 7). This is evidence of nuclear stress, and progressive metaplastic changes. Nuclear ploidy changes were also seen in tissues stored in room temperature (data not shown).

**Figure 8:**
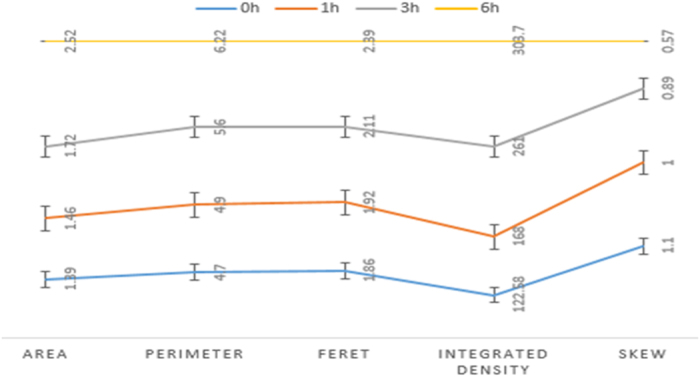
Plot of quantitative nuclear morphometry Progressive changes (increase in optical density, as well as dysplastic changes of nuclear shapes) were highly significant at the 6 hr time point, in comparison with lesser times of storage (p < 0.001, ANOVA).

Numerous morphometric parameters were assayed by ImageJ for quantitative nuclear changes during 6 hrs of storage of kidneys at 4 ^o^C (Fig. 8). These revealed progressive swelling of the nuclei, increase in the integrated density (also corroborated by the nuclear specific staining), as well as diminution in the centroid parameter, suggesting loss of normal rounded appearance of the nuclei.

## Discussion

Kidney transplantation is curative for terminal renal disease (ESRD). It is well recognized that preserving the function of the transplanted kidney in the post transplantation period is critical in terms of long term outcome^10^. The result of the present study shows that tubular epitheliocytes show disruption in cellular architecture as soon as 3 hrs post-storage ex vivo. While these observations may be a histological artefact, the chances are less likely as this was a heterogeneous presentation in a field which contained normal tubular epitheliocytes as well. Most remarkably, nuclear changes were seen as early as 1 hour after storage in the cold. Normally, most tubular epitheliocytes are open faced, uncondensed heterochromatin, suggestion of nuclear active metabolic conditions. However, just one hour after storage, several nuclei of both proximal and distal tubules appeared condensed in appearance. We have also performed numerous other assays (comparison with storage in Celsior, uniform fixation and staining conditions, as well as using cancer tissues with known ploidy changes) to clinch evidence that the changes are not random but a true biological event.

We have assayed numerous morphometric parameters suggestive of nuclear condensation and disruption of nuclear morphology. Nuclear swelling was observed; it may have resulted from osmotic defects due to the storage solution. But it is known that UWS prevents tissue edema, and thus this observation may be reflective of cytoskeletal disruption in the nuclei. Our observations were not limited to renal tissues stored in the cold. Tissues stored at room temperature in UWS and examined after 6 hours of storage showed nuclear condensation similar to tissues stored in the cold, thus suggesting that the temperature of storage may not be the contributing reason to nuclear ploidy changes. Increasing the storage temperature to ambient conditions may raise the possibility of restoring the metabolic state of the cell to near-physiological conditions but these still cannot alter the nuclear genomic changes. Though apparently these results seem intriguing, our preliminary observations probably suggest an underlying mechanistic basis for the nuclear condensation viz, factors other than metabolic coupling with ATP are probably important for maintaining nuclear integrity. One way of examining this aspect may be to perfuse the organ ex vivo during storage and thereafter re-examine these mechanisms. In fact, numerous current technology are being advanced with the feasibility of storage of solid organs during *in vitro* conditions with continuous perfusion with blood warmed at 37 °C^25^.

Because of our uniform storage conditions in UWS, the nuclear changes are likely attributable to the cold conditions of storage and thus lesions may be induced due to hypothermia. Thus, these changes may actually be reflective of an ongoing genomic insult due to the cold storage condition *in vitro*, and which probably persists after transplantation. Tubular injury is the reason for renal failure in several conditions and this likely results in delaying of recovery of renal function post-transplantation. The results of our present study also prompt to examine other renal microarchitecture and whether they may be damaged by tepid storage. This also provides rationale for designing more effective solutions or exploring the possibility of storage in subnormothermic or room temperatures^11^,^12^, which may not cause genomic nuclear insults during ex vivo storage. Storage of other solid organs have shown the effect of warm temperature storage as maintaining the organ physiological function^26^,^27^. The results of the current study do not support that storage in ambient conditions in UWS does not prevent the nuclear changes of tubular epitheliocytes. Our preliminary data provides objective evidence of lesions caused by cold storage in kidneys and provides precedence for examining the beneficial effect of modulation of storage conditions, including composition of storage solutions for efficient tissue preservations.

### Highlights

1. Distinct nuclear changes occur in tubular epitheliocytes with progressive *in vitro* storage of mammalian kidneys *in vitro*.
2. This may call for storage at ambient room temperatures of organs to prevent cellular damage and prevent delayed graft non-function.

## Additional Information

**How to cite this article**: Sun, H. *et al.* Short term ex vivo storage of kidneys cause progressive nuclear ploidy changes of renal tubular epitheliocytes. *Sci. Rep.* **5**, 10341; doi: 10.1038/srep10341 (2015).
